# Incorporating the Gut Microbiome in the Risk Assessment of Xenobiotics and Identifying Beneficial Components for One Health

**DOI:** 10.3389/fmicb.2022.872583

**Published:** 2022-05-04

**Authors:** Antonis Ampatzoglou, Agnieszka Gruszecka-Kosowska, Alfonso Torres-Sánchez, Ana López-Moreno, Klara Cerk, Pilar Ortiz, Mercedes Monteoliva-Sánchez, Margarita Aguilera

**Affiliations:** ^1^Department of Microbiology, Faculty of Pharmacy, University of Granada (UGR), Granada, Spain; ^2^Centre of Biomedical Research, Institute of Nutrition and Food Technology “José Mataix” (INYTA), UGR, Granada, Spain; ^3^Department of Environmental Protection, Faculty of Geology, Geophysics, and Environmental Protection, AGH University of Science and Technology, Kraków, Poland; ^4^IBS: Instituto de Investigación Biosanitaria ibs., Granada, Spain

**Keywords:** one health, gut microbiome, xenobiotics, microbiota disrupting chemicals, next-generation risk assessment, antimicrobial resistance, next-generation probiotics

## Abstract

Three areas of relevance to the gut microbiome in the context of One Health were explored; the incorporation of the microbiome in food safety risk assessment of xenobiotics; the identification and application of beneficial microbial components to various areas under One Health, and; specifically, in the context of antimicrobial resistance. Although challenging, focusing on the microbiota resilience, function and active components is critical for advancing the incorporation of microbiome data in the risk assessment of xenobiotics. Moreover, the human microbiota may be a promising source of beneficial components, with the potential to metabolize xenobiotics. These may have possible applications in several areas, e.g., in animals or plants for detoxification or in the environment for biodegradation. This approach would be of particular interest for antimicrobials, with the potential to ameliorate antimicrobial resistance development. Finally, the concept of resistance to xenobiotics in the context of the gut microbiome may deserve further investigation.

## Introduction

The microbiome, a characteristic microbial community occupying a reasonably well-defined habitat with distinct physio-chemical properties, encompasses the microorganisms involved (microbiota), as well as their structural elements, metabolites, and surrounding environmental conditions (Berg et al., 2020). One Health (OH) is the holistic methodology of transdisciplinary cooperation to improve human, animal, plant, and environmental health simultaneously, and its adoption is continuously expanding (Centers for Disease Control and Prevention, 2020; Bronzwaer et al., 2021). Due to their functional potential and considering their associations with a range of diseases, microbiomes are key elements in the OH framework (Comitato Nazionale per la Biosicurezza, le Biotecnologie e le Scienze della Vita [CNBBSV], 2019; Merten et al., 2020). Their significance is partly due to pathogenic and commensal microbial transfer between humans, animals, and the environment and the human microbiome stands out with regards to its interactions with environmental and dietary chemicals that affect human health outcomes (Trinh et al., 2018). Of particular interest are the contact and mutual influence between the human gut microbiome (GM) and exogenous toxic chemicals, xenobiotics, focusing on their fate, metabolism, and toxicity (National Academies of Sciences, Engineering, and Medicine [NASEM], 2018; Abdelsalam et al., 2020).

Among xenobiotics, endocrine disrupting chemicals (EDCs) are especially important, and they have been associated with metabolic disorders, such as obesity, as well as with changes in the GM (Gálvez-Ontiveros et al., 2020; Aguilera et al., 2021). Recently the concept of microbiota disrupting chemicals (MDCs) has been proposed, which comprise EDCs and other xenobiotics with potential to alter the gut microbiota’s composition and metabolism (Aguilera et al., 2020) via food ingestion, e.g., bisphenols and parabens (Andújar et al., 2019; Monteagudo et al., 2021; Robles-Aguilera et al., 2021).

The interactions between MDCs and the GM are complex. This is partly because multiple general mechanisms are involved including; direct effects of the MDC on the microbiome; altered epithelial-barrier functions (affecting uptake or excretion of MDCs); direct chemical transformations of MDCs; secondary transformation of host-generated metabolites (e.g., deconjugation by β-glucuronidases), and; altered expression of host-tissue metabolic enzymes and pathways (e.g., in the liver *via* microbial signaling molecules) (Ulluwishewa et al., 2011; Patterson and Turnbaugh, 2014; Peterson and Artis, 2014; Kelly et al., 2015; Selwyn et al., 2015, 2016; Claus et al., 2016; Spanogiannopoulos et al., 2016; National Academies of Sciences, Engineering, and Medicine [NASEM], 2018). Although these interactions can decrease MDC exposure and toxicity effects, they can also increase them. For example, several bacterial phyla in the human GM can produce azoreductases, which have been shown to reduce azo dyes that are common in foods into mutagenic and carcinogenic aromatic amines (Rafii et al., 1990; Xu et al., 2007). Overall, the role of these complex interactions in modifying human susceptibility to MDCs is beginning to be elucidated.

Risk assessment (RA) is the science-based component of the food safety risk analysis framework, alongside risk management and risk communication. RA comprises; hazard identification; hazard characterization; exposure assessment, and; risk characterization (Codex Alimentarius Commission, 1999; Regulation (EC) No 178/2002, 2002). Traditionally, xenobiotic RA relies on data from animal experiments, human trials and/or human observational/epidemiological studies. Importantly, the extrapolation of this data across species or studied populations is not without challenge, partially due to GM variability and the complexity of MDC/GM interactions (National Academies of Sciences, Engineering, and Medicine [NASEM], 2018). Thus, the need for the incorporation of the GM in food safety RA of xenobiotics is well-justified (Merten et al., 2020) and by extension to MDCs.

Another area of relevance to the GM in the context of OH is the identification of beneficial taxa and derived components (e.g., enzymes and biocompounds) in the GM and their potential application. In this context, toxicomicrobiomics, which study the aforementioned microbiome-xenobiotic/MDC interactions, along with culturomics, which aim to cultivate components of the human GM through the use of optimized selective and/or enrichment culture conditions coupled with metagenomic taxa identification, can shed light on the microbiome’s capacity to metabolize xenobiotics (Aziz, 2018; Lagier et al., 2018; Comitato Nazionale per la Biosicurezza, le Biotecnologie e le Scienze della Vita [CNBBSV], 2019; Abdelsalam et al., 2020; López-Moreno et al., 2022) and by extension MDCs. Thus, these approaches can help identify GM components with beneficial effects under OH, for example detoxification activity (López-Moreno et al., 2021b) or next-generation probiotics (NGPs) (López-Moreno et al., 2021a).

A third area of relevance is antimicrobial resistance (AMR). Undoubtedly, AMR is an important OH issue, with the major contributor being the misuse of antibiotics (World Health Organization, 2015, 2021; O’Neill, 2016). Moreover, the GM has previously been considered as a reservoir for antibiotic resistance genes (Gibson et al., 2015; Anthony et al., 2021). Non-antibiotic antimicrobials, including MDCs triclosan and parabens, commonly used as preservatives in foods, food contact materials (FCMs) and personal care products (Soni et al., 2001, 2002, 2005; Cosmetic Ingredient Review Expert Panel, 2008; Halden et al., 2017), may also contribute to AMR (Scientific Committee on Consumer Safety [SCCS], 2010). This is because some resistance mechanisms are common to both biocidal MDCs and antibiotics, for example, the former may; exert selective stress leading to the expression of bacterial resistance mechanisms and their dissemination, and/or; maintain mobile genetic elements carrying genes involved in antibiotic cross-resistance (Scientific Committee on Emerging and Newly Identified Health Risks, 2009). Therefore, due to their detoxification potential certain GM taxa may become of particular interest in the context of such antimicrobials.

**FIGURE 1:**
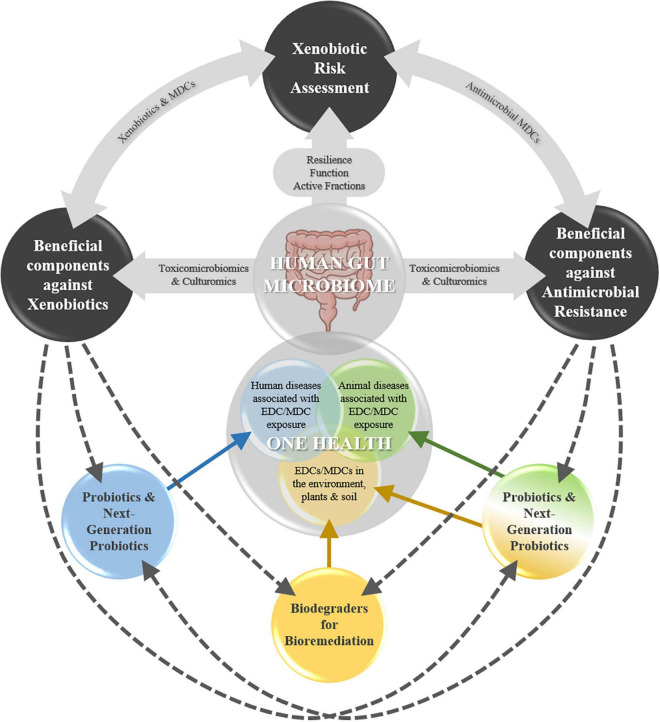
Three interlinked areas of relevance to the human gut microbiome (GM) in the context of One Health (OH); incorporation of the GM in food safety risk assessment of xenobiotics; identification and application of beneficial GM taxa and components (e.g., enzymes and bioactive compounds) to various areas under OH, and; specifically, in the context of antimicrobial resistance. EDC, endocrine disrupting chemicals; MDC(s), microbiota disrupting chemicals.

This perspective discusses these interlinked areas of relevance to the GM in the context of OH (Figure 1). The first area relates primarily to human health. However, depending on the output of the RA and the antimicrobial or not nature of the xenobiotic, the other two areas are highly relevant to holistic xenobiotic risk management.

## Incorporating the Gut Microbiome in Food Safety Risk Assessment of Xenobiotics Under One Health

GM variability adds layers of complexity to the already intricate interactions between MDCs and health. The observed differences in the GM have been attributed to multiple factors including age, antibiotic use, diet, disease state, environmental exposures, exercise, genetics, geography, pregnancy status, sex, socioeconomic status, and surgical interventions (Dethlefsen and Relman, 2011; Koren et al., 2012; Yatsunenko et al., 2012; Markle et al., 2013; Goodrich et al., 2014; O’Sullivan et al., 2015; Tremaroli et al., 2015; Levin et al., 2016; Mar et al., 2016; National Academies of Sciences, Engineering, and Medicine [NASEM], 2018). Moreover, these factors may only explain a small fraction of the total GM variation (Falony et al., 2016; Comitato Nazionale per la Biosicurezza, le Biotecnologie e le Scienze della Vita [CNBBSV], 2019). Importantly, due to this variability, observations of microbiome-influenced toxicities in a studied population might have little relevance to other populations with substantially different GM composition and function (Rodricks et al., 2019).

In addition, there is considerable variation between the GMs of humans and animals, due to anatomical, physiological, functional, immunological and compositional differences. Some of these have been partially overcome via the use of “humanized” animals in toxicological studies (Sonnenburg and Bäckhed, 2016). Nevertheless, extrapolation from such studies to humans still carries considerable uncertainty (Rodricks et al., 2019) and, along with the intraspecies variability, necessitates the use of uncertainty/safety factors, frequently reaching two orders of magnitude (Dorne and Renwick, 2005; Benford et al., 2018). Based on these factors, traditional RAs may overestimate or underestimate the risk associated with exposure to an MDC, partially because they do not account for its interactions with the microbiome (National Academies of Sciences, Engineering, and Medicine [NASEM], 2018; Merten et al., 2020). Consequent risk management decisions may place considerable pressure on the industry. For example, EFSA’s recent proposal to considerably reduce the tolerable daily intake for Bisphenol A (BPA) (Lambré et al., 2022), may further increase the use of bisphenol analogs in FCMs, which may also trigger dysbiosis and obesogenic phenotypes (Andújar et al., 2019; Monteagudo et al., 2021).

Although the need is clear to incorporate the GM in the RA of xenobiotics, there are additional hurdles, i.e., the fundamental requirements to; establish causation and molecular mechanisms linking phenotypes, e.g., obesity, with microbiota profiles (Fischbach, 2018), and; define what constitutes a healthy GM, which still remains elusive (Merten et al., 2020). Considering that these tasks require significant resources, it might be a useful first step to establish principles on how to evaluate the potential of xenobiotics to alter the GM.

Interestingly, a three-tier framework has recently been proposed by the Unilever Safety and Environmental Assurance Center for assessing the potential of personal care formulations to perturb the skin and oral microbiomes (Métris et al., 2021).

The first tier benchmarks new formulations against ones regarded as safe because of a long “history of safe use” (HoSU). However, this approach cannot apply directly to xenobiotics, for reasons such as their nature as contaminants or that they may not be intended to be ingested (e.g., if used in FCMs). Moreover, it is challenging to establish robust links between GM, cumulative exposure and resulting adverse effects (Ortiz et al., 2022). Nevertheless, evidence has been compiling in recent years on the impact of several contaminants and groups of xenobiotics, including pesticides, bisphenols, phthalates, metals, triclosan, parabens and polybrominated diphenyl ethers, on human and animal gut microbiomes (Aguilera et al., 2020). As it expands, this evidence could potentially serve as an early cross-reference tier which would raise initial concerns, depending on the nature and chemical structure of a xenobiotic under RA.

The second tier focuses on microbiome resilience. Other authors highlighted resilience, along with resistance to perturbation, as a key feature of healthy microbiomes, attributed to their rich and diverse metabolic pathways (Lloyd-Price et al., 2016; McBain et al., 2019; Cheng et al., 2022). Importantly, this tier assesses risk in relative terms. Thus, it circumvents the need to define the healthy microbiome, since it is only concerned about the return to its baseline state, independently of whether healthy or desirable. Of course, the length of exposure of the microbiome to the potential perturbator would be a critical consideration. Overall, however, this tier could be a reasonable approach to screen MDCs based on the resilience of the GM under various experimental approaches, extending from “humanized” animals (National Academies of Sciences, Engineering, and Medicine [NASEM], 2018) to *ex vivo* and *in vitro* models, such as simulator of the human intestinal microbial ecosystem (SHIME) (Van den Abbeele et al., 2012), minibioreactor arrays (Auchtung et al., 2015) and multi-compartment microfluidic-based gut-on-chip systems (De Gregorio et al., 2020; Signore et al., 2021).

Finally, the third tier makes use of next-generation sequencing microbiome data in relation to host health status. This requires further development, is the most challenging tier and is, certainly, relevant to the RA of xenobiotics in the context of the GM. Métris et al. (2021) highlighted the importance of microbiome function over composition. This is not surprising, given that compositional variation might not necessarily impart key functional differences due to functional redundancy (Tian et al., 2020). Regarding research methodology, metatranscriptomics, is an established approach to focus on the functional taxa in the microbiome. More recent methodologies, however, have combined flow cytometry with omics technologies to characterize active microbial fractions in the GM, revealing a number of taxa underrepresented by traditional 16S rRNA metagenomics (Peris-Bondia et al., 2011; Maurice et al., 2013). These approaches are likely to offer valuable insights in the extrapolation of this tier’s approach to the GM, especially in the pursuit for key species or other types of biomarkers associated with host health or disease, which will be crucial for the incorporation of the GM in the RA of MDCs.

## Identifying Beneficial Gut Microbiome Taxa and Other Components and Applying Them Under One Health

Taxa culturing strategies, in the context of the interactional triangle between EDCs (obesogens)-gut microbiota (dysbiosis vs. eubiosis)-human health (obesity vs. leanness), are key in obtaining and selecting strains (associated with pro-obesity and antiobesity phenotypes) with potential use as NGPs (López-Moreno et al., 2021a). The latter, unlike traditional probiotics, do not have a defined HoSU, and are thus subjected to more stringent regulatory requirements (O’Toole et al., 2017). Nevertheless, strains isolated from the human gut could more readily be used under OH, e.g., as probiotics for animals, plants, and environmental protection and bioremediation. Recent work has demonstrated that toxicomicrobiomics and culturomics are promising in exploring the potential of human GM taxa to metabolize obesogenic MDCs and selecting species able to tolerate or biodegrade BPA (López-Moreno et al., 2021b,^2022^). Thus, similar approaches could be used going forward to explore the human GM as a source of beneficial microbes (NGPs), enzymes, and bioactive compounds linked to MDC detoxification or biodegradation, with various potential applications under OH (Figure 1).

## Gut Microbiome and Antimicrobial Resistance Under One Health

MDCs, such as triclosan and parabens, contribute to the AMR issue, primarily through resistance development against themselves (self-resistance), but also potentially through development of cross-resistance against antibiotics (Ribado et al., 2017). Although the evidence supporting cross-resistance development *in situ* is not conclusive (Scientific Committee on Consumer Safety [SCCS], 2010), the potential contribution of MDCs to AMR and their mechanisms merit further data compilation (Valkova et al., 2002; Hughes et al., 2020; Rozman et al., 2021). Moreover, given that antimicrobial MDCs would likely have higher potential to alter and perturb microbiomes (compared to non-antimicrobial xenobiotics), they have been proposed as candidate chemicals in investigations that would built our understanding around the xenobiotic-microbiome interactions in the context of xenobiotic RA (National Academies of Sciences, Engineering, and Medicine [NASEM], 2018).

Nevertheless, even non-antimicrobial xenobiotics may pose resistance development issues in the context of the GM, as exposure to them may apply a selective pressure in favor of microbial taxa with specific enzymatic arsenals and metabolic pathways. For example, López-Moreno et al. (2022), associated BPA exposure and the obese phenotype in children to higher BPA biodegradation potential in their GM. Moreover, they reported that BPA-resistant strains isolated from human gut microbiota exhibited xenobiotic biodegradation and antimicrobial effects linked to polyketide biosynthesis (Torres-Sánchez et al., 2021). Therefore, in the presence of BPA, these strains may further modulate the composition and function of the human gut microbiota, potentially reducing GM diversity and inducing dysbiosis and adverse metabolic effects (Aguilera et al., 2020). The mechanisms, via which gut microbiome taxa may be affected by non-antibacterial MDCs, potentially leading to dysbiosis, could include growth inhibition or promotion and metabolism modulation (Lindell et al., 2022). For example, several artificial sweeteners, spice extracts and food dyes have been shown to inhibit the growth of specific bacterial strains *in vivo*, while certain natural xenobiotics and food additives appear to promote the growth of other strains under similar conditions, likely acting as nutrient sources (Pan et al., 2012; Bello González et al., 2016; Lu et al., 2017; Wang et al., 2018; Ruiz-Ojeda et al., 2019; Frame et al., 2020). Additionally, an alkaloid found naturally in coffee, trigonelline, has shown potential to alter the metabolism of a common human gut commensal *in vivo* (Anwar et al., 2018). Although limited, this evidence suggests that the potential for xenobiotic resistance development, in the context of the GM, may warrant further consideration and research, beyond antimicrobial resistance.

Overall, applying GM taxa and biocompounds able to metabolize antimicrobial MDCs to crosscutting areas under OH could potentially ameliorate AMR pressure (Figure 1).

## Conclusions

• Focusing on the GM’s resilience circumvents some of the RA challenges. Moreover, looking at function, rather than composition, and exploring the active components of the GM can help establish specific biomarkers, necessary for incorporating the GM in the RA of xenobiotics.
• The human GM may be a promising source of beneficial microbes (i.e., probiotics, NGPs and biodegraders), enzymes, and bioactive compounds, with the potential to metabolize xenobiotics. These may have potential applications in various areas under OH.
• Applying human GM components, able to metabolize antimicrobial MDCs under OH could also help ameliorate the global risk of AMR development.
• The potential for xenobiotic resistance development, in the context of the GM, may warrant further consideration.

## Data Availability Statement

The original contributions presented in this perspective are included in the article; further inquiries can be directed to the corresponding authors.

## Author Contributions

AA: conceptualization, original draft preparation, review, and editing. MA: conceptualization, review, and editing. AG-K, AT-S, AL-M, KC, PO, and MM-S: review and editing. All authors have read and agreed to the published version of the manuscript.

## Conflict of Interest

The authors declare that the research was conducted in the absence of any commercial or financial relationships that could be construed as a potential conflict of interest.

## Publisher’s Note

All claims expressed in this article are solely those of the authors and do not necessarily represent those of their affiliated organizations, or those of the publisher, the editors and the reviewers. Any product that may be evaluated in this article, or claim that may be made by its manufacturer, is not guaranteed or endorsed by the publisher.
